# Real-world clinical outcomes of cefiderocol therapy in the Veterans Health Administration, 2019–2022

**DOI:** 10.1017/ash.2023.338

**Published:** 2023-09-29

**Authors:** Eva Amenta, Barbara Trautner, David Ramsey, Andrew Chou

## Abstract

**Background:** Cefiderocol is a novel siderophore cephalosporin with broad-spectrum activity. In the CREDIBLE-CR phase 3 clinical trial examining treatment of carbapenem-resistant gram-negative infections, cefiderocol had similar clinical and microbiological efficacy compared to the best available therapy, but the mortality rate was unexpectedly higher in the cefiderocol group. We investigated the postapproval, real-world clinical outcomes of cefiderocol therapy. **Methods:** We conducted a prospective, observational study of patients who received cefiderocol for at least 2 days within the Veterans’ Health Administration (VHA) between the date of approval by the US Food and Drug Administration (FDA), November 14, 2019, and August 31, 2022. Types of infections were defined by NHSN criteria. Clinical failure was a composite outcome based on type of infection including survival (30- and 90-day mortality) and resolution of signs and symptoms of infection. Microbiologic failure was defined as culturing the same organism, as defined by the CDC NHSN, at least 7 days after the start of cefiderocol. Structured data were sourced from the VHA Corporate Data Warehouse, and each eligible episode underwent manual chart review. **Results:** During the study period, 8,763,652 patients across 132 VA medical centers received 1,142,940,842 prescriptions (not limited to antibiotics). Overall, 48 unique individuals had received cefiderocol, with 48 cefiderocol courses prescribed. Patients had a median age of 70.5 years (range, 61–75), and a median Charlson comorbidity score of 6 (range, 2–9). The most common infectious syndromes were lower respiratory tract infection in 23 (47.9%) of these 48 patients and urinary tract infection in 14 (29.2%) of these patients. The most common pathogens cultured were *P. aeruginosa* in 30 patients (62.5%), Enterobacterales in 17 patients (35.4%), and *A. baumannii* in 10 patients (20.8%). The clinical failure rate was 35.4% (17 of 48), and 15 (88.2%) of these 17 patients died within 3 days of clinical failure. The 30-day and 90-day microbiologic failure rates were 33.3% (16 of 48) and 45.8% (22 of 48), respectively. The 30-day and 90-day all-cause mortality rates were 27.1% (13 of 48) and 45.8% (22 of 48), respectively (Table 1). **Conclusions:** Our study cohort included older individuals with multiple comorbidities who were treated with cefiderocol mainly for lower respiratory tract and urinary tract infections, with *Pseudomonas aeruginosa* as the main causative pathogen. Clinical and microbiologic failure were seen in>30% of patients, and >40% of these patients died within 90 days. These data contribute to the growing body of literature on the real-world use of cefiderocol and provide outcome data on clinical failure, microbiologic failure, and mortality.

**Figure:**
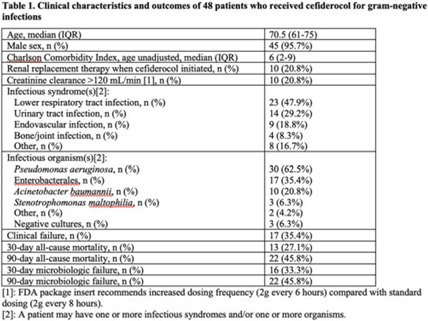

**Financial support:** This study was supported by the Department of Veterans Affairs Clinical Science Research and Development (VA CSRD grant no. IK2 CX001981).

**Disclosures:** None

